# *Bifidobacterium infantis—*a key (late) colonizer of the infant gut?

**DOI:** 10.1128/msphere.00851-25

**Published:** 2026-01-26

**Authors:** Martin F. Laursen

**Affiliations:** 1 National Food Institute, Technical University of Denmark 5205, Kongens Lyngby, Denmark; University of Michigan Medical School, Ann Arbor, Michigan, USA

**Keywords:** bifidobacteria, probiotics, intestinal colonization

## Abstract

Human milk oligosaccharide (HMO)-degrading *Bifidobacterium* species are key early colonizers of the gut and influence gut and immune maturation. Loss of these taxa, particularly *Bifidobacterium infantis*, in many industrialized populations has raised concern. O’Brien et al. showed that supplementation with *B. infantis* EVC001 in exclusively breastfed U.S. infants aged 2–4 months leads to rapid and abundant colonization that persists 1 month after supplementation, demonstrating effective colonization beyond the neonatal period (C. E. O'Brien, S. A. Frese, K. Cernioglo, K. Damian-Medina, et al., mSphere e00518-25, 2025, https://doi.org/10.1128/msphere.00518-25). These findings align with observational cohort data showing that *B. infantis* can overcome priority effects and dominate the gut microbiome in breastfed infants by 2-3 months of age. Key questions remain regarding colonization in mixed- or formula-fed infants, the HMO thresholds required to sustain dominance during milk- and complementary feeding, and the critical developmental windows of *B. infantis* colonization for beneficial immune effects. Ongoing clinical trials with *B. infantis* will further clarify its role in disease prevention.

## COMMENTARY

Human milk oligosaccharide (HMO)-degrading *Bifidobacterium* species are key early colonizers of the infant gut closely associated with breastfeeding and instrumental in gut and immune maturation (1, 2). Their abundance in early life is inversely associated with the later development of atopic disease (3–5). Consequently, the widespread loss of these taxa—particularly the highly milk-adapted *Bifidobacterium longum* subsp. *infantis* (*B. infantis*)—in the infant gut microbiomes of many industrialized societies is a cause for concern (4).

O’Brien et al. (6) examined the effect of *B. infantis* EVC001 supplementation in exclusively breastfed U.S. infants aged 2–4 months, a population previously reported to lack this subspecies (7). Prior studies using this strain (8, 9) or other *B. infantis* strains (10, 11) have shown that neonatal supplementation produces rapid, efficient, and persistent colonization. Data are scarce, however, on supplementation initiated beyond the neonatal period (>1 month) when the gut microbiome is more established. O’Brien et al. demonstrated that rapid and high-level *B. infantis* colonization is also achievable in this later infancy window. This aligns very well with data from various observational cohorts (12, 13), showing that *B. infantis* is often a late colonizer (around 2–3 months of age) and can even take over as the dominant microbial taxon, even if the breastfed infants are abundantly colonized by other HMO-utilizing *Bifidobacterium* species (14).

In the new study of O’Brien et al., the authors notably showed that the high abundance of *B. infantis* persisted at least 1 month after supplementation ceased—importantly, a setting where the infants remained exclusively breastfed. As the prototype HMO degrader, *B. infantis* is optimally suited to colonize and persist in the gut of breastfed infants (15). It remains unclear, however, whether the subspecies can colonize as effectively in mixed-fed or formula-fed infants, who constitute a large proportion of infants worldwide (16). Given its specialization for HMO utilization, efficient colonization and persistence in formula-fed infants seem unlikely unless formulas are supplemented with HMOs, since HMO-supplemented formulas have been shown to partially restore *Bifidobacterium* levels toward those observed in breastfed infants (17, 18).

From a microbial ecological perspective, it is of interest to understand when ingested HMO concentrations fall below the threshold needed to sustain *B. infantis* dominance. For example, is there a tipping point in the breastmilk:formula ratio that no longer favors *B. infantis* dominance? Can *B. infantis* abundance be maintained during complementary feeding—when solid foods are introduced—if infants remain partially breastfed or receive HMO supplementation? At what stage do the competitive advantages conferred by HMO utilization cease to support its persistent and abundant colonization?

From a clinical perspective, key questions remain about critical developmental windows during which robust colonization by *B. infantis* (or other infant-associated *Bifidobacterium* species) is necessary to confer protection against immune-related diseases and at which point in time colonization would no longer produce durable immune benefits.

O’Brian et al. also investigated different doses of *B. infantis*, showing equally good colonization at all tested doses (4 × 10^9^, 8 × 10^9^, and 1.8 × 10^10^ CFU/day). While this shows that the lower dose is adequate, it does not establish the minimum required dose that ensures successful colonization, which would be of ecological interest. In fact, the authors provide anecdotal evidence of potential inter-personal transmission from an infant in the supplemented group to an infant in the placebo group, likely due to the two mothers reporting spending substantial time together. While this indicates that much lower inoculation sizes are adequate to establish robust colonization, the infant-to-infant transmission and colonization dynamics are not well understood. Nonetheless, it remains important to understand how/why some populations might have lost *B. infantis*.

Importantly, *B. infantis* remains prevalent in infants from many nonindustrialized settings (e.g., Bangladesh, Gambia, and Tanzania [12, 19]) with a high breastfeeding prevalence and has been observed to dominate the gut microbiome of breastfed infants in some industrialized cohorts (e.g., Denmark and Israel [13, 14, 20]). Even within the United States, rural versus urban lifestyles have been associated with differences in *B. infantis* prevalence and abundance (3). The reasons for these geographic and population differences are not fully resolved, but lack of historical breastfeeding practices across generations, potentially compounded by widespread antibiotic use and cesarean delivery, likely contributes to community-level depletion of *B. infantis* (12). This illustrates the point that the necessity for *B. infantis* supplementation is likely to be population or subpopulation specific.

Several ongoing clinical trials are evaluating the health effects of early *B. infantis* supplementation, including the GPPADSINT1A study (NCT04769037) assessing prevention in infants genetically predisposed to type 1 diabetes, an intervention trial in infants at risk for atopic dermatitis (NCT04662619), and the BEGIN study (NCT06452199) examining supplementation in healthy infants with broader endpoints, such as growth, immune function, and inflammation. Results from these and other future studies are needed to clarify the role of *B. infantis* in disease prevention. Meanwhile, the data presented by O’Brien et al. complemented prior work and reinforce that rapid, efficient, and persistent colonization by a key early-life *Bifidobacterium* species is achievable in exclusively breastfed infants, even when supplementation begins beyond the neonatal period.
